# Bone, fat, and muscle interactions in health and disease

**DOI:** 10.20945/2359-3997000000550

**Published:** 2022-11-10

**Authors:** Mayra Macena Gomes, Maisa Monseff Rodrigues da Silva, Iana Mizumukai de Araújo, Francisco José Albuquerque de Paula

**Affiliations:** 1 Universidade de São Paulo Faculdade de Medicina de Ribeirão Preto Departamento de Medicina Interna São Paulo SP Brasil Departamento de Medicina Interna, Faculdade de Medicina de Ribeirão Preto, Universidade de São Paulo, São Paulo, SP, Brasil

**Keywords:** Osteoporosis, obesity, diabetes mellitus, energy metabolism, mineral metabolism

## Abstract

Energy metabolism is a point of integration among the various organs and tissues of the human body, not only in terms of consumption of energy substrates but also because it concentrates a wide interconnected network controlled by endocrine factors. Thus, not only do tissues consume substrates, but they also participate in modulating energy metabolism. Soft mesenchymal tissues, in particular, play a key role in this process. The recognition that high energy consumption is involved in bone remodeling has been accompanied by evidence showing that osteoblasts and osteocytes produce factors that influence, for example, insulin sensitivity and appetite. Additionally, there are significant interactions between muscle, adipose, and bone tissues to control mutual tissue trophism. Not by chance, trophic and functional changes in these tissues go hand in hand from the beginning of an individual’s development until aging. Likewise, metabolic and nutritional diseases deeply affect the musculoskeletal system and adipose tissue. The present narrative review highlights the importance of the interaction of the mesenchymal tissues for bone development and maintenance and the impact on bone from diseases marked by functional and trophic disorders of adipose and muscle tissues. Arch Endocrinol Metab. 2022;66(5):611-20

## INTRODUCTION

In the evolution of species, the development of mesenchymal tissues has endowed living beings with special skills for survival in adverse environments such as those with food shortages or low temperatures and situations requiring physical confrontation against aggressors or movements with different degrees of sophistication and speed. No matter the type of mesenchymal tissue – soft (fat and muscle) or hard (bone) – it is possible to easily identify the main function of each of these important body components. In addition to mechanical functions, these tissues have clear storage roles, *i.e.*, storage of proteins by muscle, lipids by fat, and minerals by bone. The metabolic importance of the muscle tissue in adjusting glucose or free fatty acid uptake depending on feeding status has long been recognized (1). However, more time was required for the understanding of the actual importance of the adipose tissue as a site of metabolic regulation beyond its function of energy storage. The list of additional functions of the adipose tissue includes thermal insulation, thermogenesis, mechanical impact absorption, lubrication (joints), and cosmetic (2). In this regard, it is worth noting that only in this century was due attention given to aspects other than mineral ones in the study of bone metabolism, allowing the recognition of the importance of energy consumption by bone tissue and participation of bone tissue as an active site in controlling energy modulation. A non-trivial aspect that naturally connects mesenchymal tissues is their same embryonic origin from somites derived from paraxial (muscle and bone) and lateral (adipose) mesoderm (3). Not by chance, nutritional disorders and metabolic diseases like obesity and diabetes mellitus (DM) have a high impact on the structure, function, development, and maintenance of bone mass (4,5). In recent decades, research advances such as the development of imaging technologies, molecular biology, and genetic engineering have allowed unprecedented progress in the recognition of the functional plasticity of mesenchymal tissues. In this process, the complex mutual endocrine interaction between mesenchymal tissues has become increasingly clear, *i.e.*, one tissue stimulating or inhibiting the trophism of the other (6). Thus, there is great interest in unveiling the participation of muscle and adipose tissue in the development of bone damage in several diseases.

For the preparation of this narrative review, we performed a PubMed search using the following keywords: bone AND muscle, bone AND adipose tissue, osteoporosis, obesity, weight loss, and sarcopenia. We then made a careful selection of the articles which were to be included in the manuscript. This narrative review has been specially designed with these considerations in mind to present the reader with an up-to-date view on this topic.

## ADIPOSE TISSUE AND SKELETON INTERACTION

No other element in the human body surpasses the adipose tissue’s ability to expand. Thus, adipose tissue plays a crucial role in determining body weight. Body weight, in turn, has a strong positive association with bone mass (7). Despite conflicting data regarding which tissue – muscle or fat – has a greater influence on bone mass, there is no doubt that both play a strong role in the development and maintenance of bone mass (8). During the growth phase in childhood and adolescence, the concomitant development of mesenchymal tissues suggests a possible mutual and positive interrelationship between them (9). Along the same lines, it is possible to assume a non-linear relationship between the adipose tissue and the other mesenchymal tissues. During aging and in diseases such as DM and obesity, the expansion of adipose tissue is not associated with muscle or bone strengthening or performance (2,10–12). From a metabolic point of view, it is well known that adipose tissue can exert either a beneficial effect by favoring insulin sensitivity or a detrimental effect by producing an environment that generates insulin resistance (13). Likewise, it is quite possible that, in some circumstances, adipose tissue can have a negative or positive effect on the skeleton. In the last two decades, several groups have been dedicated to deciphering the mechanisms of influence of adipose tissue on bone (14,15).

In recent years, there has been a marked advance in knowledge about possible mechanisms involved in the intricate interaction between bone and adipose tissue (14). The current scenario shows that this process comprehends a complex communication network involving the participation of endocrine factors originating in adipocytes that act directly on their receptors expressed in bone cells (*e.g.*, leptin and adiponectin in osteoblasts and chemerin in osteoclasts) (Table 1), but also indirectly through hypothalamic activation of the sympathetic system. However, these hormones’ central and peripheral actions do not always converge. For example, pioneering studies by Karsenty and Khosla have shown by various means that leptin has a central action, stimulating the sympathetic nervous system (14,16). Consequently, there is inhibition of osteoblastic activity and increase in osteoclastic action, resulting in a negative balance in bone remodeling, which – ideally – should be for renewal and maintenance of bone mass in adults. On the other hand, studies indicate that leptin acts directly on osteoblasts, increasing their activity (17). An additional aspect that complicates the understanding of the final effect of leptin is the occurrence of resistance to its action. However, there is no evidence of leptin resistance in terms of its effects on bone.

**Table 1:** Endocrine interactions between adipose and bone tissues

Factor	Origin	Effects
Leptin	AD	Direct: increase in osteoblastogenesis (2). Indirect: increased sympathetic tone and blockade of osteoblastogenesis (2).
Adiponectin	AD	Direct: blockade of osteoblastogenesis (2). Indirect: reduction of sympathetic tone and induction of osteoblastogenesis (98).
RANKL	AD, OB	Stimulation of OC differentiation and activation (2).
Lipocalin	AD, OB	Osteogenic and anti-adipogenic action (14).
Chemerin	AD	Increase in adipogenesis at the expense of osteoblastogenesis (2), although animal models show osteogenic differentiation and bone formation (99).

AD: adipocytes; OB: osteoblasts; OC: osteoclasts; OST: osteocytes; CD: chondrocytes.

Decreased circulatory levels of leptin are a common finding in young women with hypothalamic amenorrhea. A previous study evaluated metreleptin therapy in women with low body weight and documented hypoleptinemia. Those who completed 2 years of therapy exhibited significant increases in lumbar spine bone mineral density (BMD) (4%), as well as in lumbar spine bone mineral content (6%). Additionally, a reduction was observed in serum levels of carboxy-terminal cross-linked telopeptide of type I collagen (CTX) and N-terminal procollagen type I propeptide (P1NP) during the second year of treatment (18).

The ability to withstand mechanical impact, one of the main properties of bone, depends on a set of factors. One of these factors is the amount of bone mass, which can be easily estimated using bone densitometry. However, other attributes (grouped as bone quality parameters) conferring strength to bone tissue still lack standardized estimation. Predisposition to fracture also occurs in some situations where bone mass is not particularly affected (19). Curiously, the most frequent situations in which this occurs are associated with significant changes in adipose tissue, including obesity, type 2 DM, and hypercortisolism (20–22). These situations are addressed below.

## OBESITY AND INSULIN RESISTANCE

Obesity is the most frequent metabolic disorder affecting humans. The World Health Organization (WHO) defines obesity as a state of fat accumulation that poses a risk to health in individuals with a body mass index (BMI) ≥ 30 kg/m^2^. The prevalence of obesity varies among countries and is reported at 3.7% in Japan (23) and 25.9% in Brazil (24). Obesity reflects the body’s remarkable ability to store excess energy, with the subcutaneous adipose tissue being the ideal storage site. Under conditions of continuing positive balance between energy consumption and expenditure, lipids are deposited at visceral sites, and when the limit in storage capacity is reached, they are exported to other tissues (25). Adipocytes fully engorged with lipids and assimilation of lipids by parts of other tissues lead to structural and functional alteration and inflammation (26). The secretory profile of the adipose tissue changes from anti-inflammatory to proinflammatory, with the production of TNF-α, IL6, and PAI1 prevailing over, for example, that of adiponectin (26,27). Metabolic abnormalities, oxidative stress, and insulin resistance add to this chronic inflammatory condition leading to type 2 DM, steatohepatitis, and cardiovascular diseases (28,29).

The impact of obesity on the skeleton may not be positive, but it is at least different from that occurring in other organs and tissues. First, bone harbors its own adipose tissue, and bone marrow adipose tissue (MAT) in humans curiously does not appear to be a site primarily geared toward fat storage under conditions of excessive energy supply (15,30). Usually, increased bone marrow adipogenesis is associated with impaired maintenance of bone mass, a situation that occurs with aging, hypercortisolism, and, paradoxically, anorexia nervosa (2,15,31,32). However, several studies have shown that insulin resistance does not correlate negatively with bone mass. Likewise, parameters strongly associated with insulin resistance, such as visceral adipose tissue (VAT) and the amount of intrahepatic lipids (IHL), are not negatively associated with bone mass (33–35). Despite a lack of consensus regarding these results, they are perfectly aligned with data established in the literature showing normal or increased bone mass in situations of insulin resistance, such as those occurring in obesity and type 2 DM. Notably, in generalized congenital lipodystrophy, a situation of severe insulin resistance, BMD is increased (36).

The initial perception that obesity was protective against fractures has changed after prospective studies have shown that individuals with obesity present fractures as frequently as those with normal weight (37). However, this finding was based on variable data that depended on the characteristics of the population, sex, age, and, in particular, fracture site. Some meta-analyses have shown a decreased risk of femoral neck fractures in obese individuals (38). A meta-analysis including cohorts from several countries has reported that obese compared with non-obese women had a lower risk of hip and distal forearm fractures but a higher risk of humeral/elbow fractures. In contrast, some studies have shown an increased risk of hip fractures in obese individuals. For example, a prospective Korean study has found a positive association between BMI and risk of hip fracture only in women with BMI above 25 kg/m^2^ (39). This finding was supported by the results of a meta-analysis indicating a positive relationship between waist circumference and hip fracture (40). A lack of consensus is also observed regarding a higher risk of vertebral fractures in obese individuals. Despite this lack of consensus, most results suggest that obesity is associated with a higher risk of peripheral fractures affecting the humerus, tibia, and ankle (37).

Several factors can be listed as responsible for the loss of bone strength in obesity and insulin resistance, such as vitamin (D) deficiency, hypothalamic hypogonadism, sarcopenia, changes in microbiota, chronic inflammation, and sedentary lifestyle (10,41–43). From a clinical perspective, it is evident that bone densitometry underestimates the risk of fracture in obesity (44). Therefore, there is need for improvement in the clinical assessment of the susceptibility to fracture in this situation. Even a more sophisticated imaging method like high-resolution peripheral quantitative computed tomography (HRpQCT) may not capture bone changes in obesity, as observed in a 2015 study by Evans and cols. (44). Trabecular bone score (TBS) is a bone texture index obtained from lumbar spine densitometry images and a predictor of fracture risk independent of BMD or bone size (45–47). This index can capture bone changes in obese individuals and may help determine risk factors for assessing fracture susceptibility in this condition. Despite significant advances in knowledge regarding the mechanisms and assessment of bone fragility in obesity, many aspects must still be unraveled. Hopefully, more options for recognizing and preventing bone fragility in obesity should emerge soon.

## DIABETES MELLITUS

According to the International Diabetes Federation (IDF), DM affected 463 million people in 2019, corresponding to a worldwide prevalence of 9% (48). The classic macroangiopathic and microangiopathic complications associated with this metabolic disease overload the countries’ health systems and are among the leading causes of chronic kidney disease, blindness, amputations, and cardiovascular diseases. Until recently, only type 1 DM was considered a secondary cause of osteoporosis. It is now recognized that type 2 DM, responsible for about 90% of DM cases, is also associated with fractures, significantly increasing the burden for health providers in terms of caring for patients with osteoporosis and DM due to the coexistence of both diseases (49,50).

As in obesity, bone mass is not negatively affected in patients with type 2 DM, with studies showing that bone mass in these patients is normal or increased (51,52). In type 1 DM, in turn, bone mass is lost in the initial phase of the disease and does not seem to be recovered later (53,54). This fact must contribute to the higher susceptibility to fractures observed in type 1 DM compared with type 2 DM. However, several other hormonal and metabolic factors (*e.g.*, IGF1 deficiency, hypoinsulinemia, and low body weight) must also play a role, considering that recent studies have shown that bone mass loss in type 1 DM is not prominent (55). Therefore, bone fragility is an additional mark of the heterogeneity among different forms of DM that are united by the occurrence of hyperglycemia.

The image linked to type 1 DM of emaciation, weight loss, and low body fat is part of the past for those who have access to new technologies and receive insulin in multiple doses or through infusion pump (56). Several studies have shown a high rate of overweight/obesity in type 1 DM (57). This new body profile of patients with type 1 DM may impose both positive and negative impacts on the skeleton, as discussed in the obesity section of this article. Recent studies have shown that bone loss in type 1 DM is not very significant (54). Therefore, as in type 2 DM, changes in bone quality must also play an important role in bone fragility in type 1 DM. Body fat distribution has been less studied in type 1 DM. Using magnetic resonance imaging, Carvalho and cols. have shown that, compared with a control group, individuals with type 1 DM with good or poor metabolic control have quantitatively similar subcutaneous adipose tissue (SAT), VAT, and IHL (55). The study also showed no differences in the amount of MAT between patients with type 1 DM and controls (54).

## ANOREXIA NERVOSA

Weight loss by self-imposition of fasting is the hallmark of anorexia nervosa, a psychiatric disease that affects mainly adolescent and young adult women. Among the psychiatric diseases, anorexia nervosa is distinguished by a high rate of death by suicide (58) and is associated with several morbidities, including osteoporosis. The fracture risk is about three times greater in patients with this condition, and low bone mass is likely an important predisposing factor (59). Therefore, the skeleton follows the wasting process that affects muscle and adipose tissues in a state of food restriction. Part of the function of muscle and adipose tissues, as storage sites for energy substrates, is to provide, respectively, amino acids and fatty acids to meet energy needs, orchestrated by the well-recognized adaptation of hypoinsulinemia and elevation of insulin counterregulatory hormones (60,61). The bone repercussions during adaptations caused by malnutrition in anorexia nervosa are well recognized, *i.e.*, growth arrest and loss of bone mass gain in the development phase and bone loss in adulthood. Hormonal abnormalities in patients with anorexia nervosa include hypothalamic hypogonadism, increased cortisol, and low levels of IGF1, insulin, and leptin (61,62).

A peculiar aspect of the wide heterogeneity of adipose tissue is the expansion of MAT under conditions of food restriction, as shown experimentally in mice fed with 30% fewer calories than controls (63). Studies in women with anorexia nervosa have shown the same pattern and demonstrated that the increase in MAT correlates negatively with bone mass (64). Thus, MAT expansion in anorexia nervosa is aligned with observations from common conditions in which osteoporosis occurs, such as aging and glucocorticoid-induced osteoporosis (65,66). Bone remodeling markers suggest a disconnect between bone formation and resorption, with studies showing a reduction in bone formation markers and an increase in markers that reflect resorption (67). Since osteoblasts and adipocytes share a common origin from the same mesenchymal stem cell, it is reasonable to consider that the direction of the differentiation occurs toward one cell lineage at the expense of the other, with adipocytes prevailing in this case (68). The role of the paracrine activity of adipocytes in determining the fate of mesenchymal stem cells under these circumstances remains to be determined.

## WEIGHT LOSS AFTER SURGICAL INTERVENTIONS IN THE GASTROINTESTINAL TRACT (BARIATRIC SURGERY AND SHORT BOWEL SYNDROME)

Given the current obesity pandemic and the frustrating limitation of clinical alternatives for obesity treatment (69,70), aggressive management through restrictive and malabsorptive surgery has emerged in recent decades as the most efficient option to mitigate morbidity and mortality of severe obesity (71). This approach has drawbacks; the loss of bone mass and increased susceptibility to fracture are among the most important.

There is a paucity of prospective data evaluating both the bone loss and metabolic benefits of bariatric surgery in severely obese individuals (72). However, a recent cross-sectional study has shown metabolic benefits obtained with bariatric surgery, which persist for 5 years after the procedure, even in patients whose BMI remains in the range of obesity. After bariatric surgery, the VAT/SAT distribution ratio and the amount of IHL were similar in individuals with obesity or normal weight. In contrast, individuals submitted to bariatric surgery present early elevation of remodeling marker of bone resorption, a pattern that is maintained for at least 5 years after surgery (72). Two previous studies, one cross-sectional (72) and the other prospective (73), have suggested precocious and sustained increased levels of the biochemical marker of bone resorption CTX. In the former, Alencar and cols. described that the groups evaluated 1 and 5 years after Roux-en-Y bypass (RYGB) showed levels of serum CTX that were, respectively, 97% and 51% higher than those in the control group (72). In a prospective study, serum CTX levels have been found to be 150% above the basal levels 5 years after RYGB (73). On the other hand, the literature shows more modest or no difference in the biochemical markers of bone formation after bariatric surgery (72,73). For instance, Alencar and cols. have also observed that, compared with a control group, individuals submitted to RYGB show similar serum osteocalcin levels both 1 and 5 years after the procedure. However, circulatory levels of P1NP were 34% higher than baseline 5 years after RYGB. These results are in line with previous data showing that RYGB has detrimental effects on bone microstructure as well as bone strength. The impact on bone after RYGB appears early in the first year and persists for at least a few years after weight loss plateaus.

Notably, bone mass measured by bone densitometry may not be an efficient way to detect bone catabolic states in individuals with severe obesity, as these individuals generally start from a high bone mass value (72). However, after bariatric surgery, the BMD/body weight ratio has been shown to be significantly lower at 5 years compared with 1 year (72). Another study has shown that MAT in adolescents undergoing bariatric surgery is increased in the lumbar spine and reduced in long bones (74). Thus, it is possible that, in addition to decreased food intake and absorption of nutrients (*e.g.*, calcium and vitamin D) and reduced serum levels of IGF1 and insulin, the increased MAT may contribute to a negative balance in the process of bone remodeling (75).

## SHORT BOWEL SYNDROME

Individuals with anorexia nervosa and those submitted to bariatric surgery experience caloric restriction through different mechanisms. In contrast, patients with short bowel syndrome (SBS) have an alternative supply of nutrients through parenteral means, allowing for part of these patients to regain their body weight. This aspect may determine significant differences between SBS and the two other conditions (anorexia nervosa and bariatric surgery) from a metabolic point of view, but from the perspective of bone, the three conditions share negative consequences on the skeleton (76). Indeed, individuals with SBS have a high prevalence of osteoporosis and hepatic steatosis (77,78). Another aspect is that the parenteral supply of nutrients in SBS not only seems to limit MAT expansion but also changes the pattern of correlation between MAT and bone mass, which in this case is positive, suggesting that – depending on the circumstances – MAT may have a positive or negative effect on the maintenance of bone mass. Finally, it is worth noting that other variables such as incretins (GLP1 and GIP) and the microbiota are potential protagonists in bone changes in SBS, although their roles have yet to be better determined (77).

## MUSCULOSKELETAL INTERACTION

Muscle and skeleton are interconnected, forming an interdependent unit in a lever system that allows movement. The mechanical interaction between both serves as a positive mutual stimulus, depending on the appropriate nutritional environment (79,80). The mechanical interaction is contemplated in the mechanostat model proposed by Frost (81). The musculoskeletal mechanical coupling is the most visible portion of the model, and its importance can be highlighted in several studies in models ranging from neural injury (82,83), simulation of weight subtraction using tail suspension (84,85), and microgravity experiments in space (86,87). In fact, osteoporosis and sarcopenia go hand in hand in different physiological situations and are particularly present in the context of aging and in various diseases (87). New evidence shows that this interaction also occurs at molecular and biochemical levels (Table 2) and opens up the possibility of therapeutic interventions that may preserve the quality of life in older age and in various diseases (88).

**Table 2:** Endocrine interactions between muscle and bone tissue

Factor	Origin	Effects
myostatin	Skeletal muscle	Myostatin has a negative effect on osteoblast differentiation and a positive effect on osteoclast formation.
IL6	Immune system cells, endothelial cells, skeletal and smooth muscle, adipocytes, pancreas, hepatocytes, microglial cells, astrocytes, and many other cell types	Muscle-derived IL6 increases bone resorption. Increase in osteoclastogenesis *in vitro* (co-culture of osteoblasts and osteoclast progenitors).
IL15	Skeletal muscle	Stimulates differentiation of preosteoclasts into osteoclasts.
Irisin	Skeletal muscle	*In vitro*, irisin stimulates osteoblastogenesis (6). In humans (postmenopausal women), there is a negative correlation between irisin level and risk of bone fracture (79).
IGF1	Liver, bone, and skeletal muscle	Paracrine action on the muscle surface and periosteum: muscle-derived IGF1 can signal osteoprogenitor cells expressing IGF1R in the periosteum to increase bone formation (79).

IL6: interleukin-6; IL15: interleukin 15; IGF1: insulin-like growth factor, IGF1R: insulin like growth factor 1 receptor.

*Myokine* is a term used generically to refer to factors secreted by muscle tissue. The first myokine to be identified was myostatin (a potent inhibitor of myocyte proliferation), with others being later identified, including irisin (which induces browning of white adipose tissue), IL8 (which induces angiogenesis), and IL15 (which reduces adipose tissue) (89–91). The musculoskeletal interaction is definitely not a one-way path, as factors derived from bone also influence muscle metabolism. Osteocalcin, a pleiotropic peptide, may have a role in muscle trophism (92). Excess FGF23, as observed in osteomalacia-inducing tumors, is distinguished by the presentation of muscle weakness and pain that is probably not explained by hypophosphatemia alone (93). Furthermore, in chronic kidney disease, FGF23 may have a deleterious effect on the myocardium (94).

## SARCOPENIA AND OSTEOPOROSIS

As mentioned above, osteoporosis and sarcopenia often coexist in elderly individuals. At first glance, it is possible to assume that a reduction in muscle activity alone results in a loss of bone maintenance due to overestimating the dependence of bone biomechanics on the muscular system. However, the picture may be more complex, and other factors can contribute to the bone fragility that settles in elderly individuals with sarcopenia. In reality, the aging process is a complex and incompletely understood occurrence. For example, osteocytes comprise 90%-95% of bone tissue cells; these are terminally differentiated cells established to remain encased in the calcified matrix for decades. Osteocytes become senescent and have their functional capacity modified by altered gene expression and change in secretome to a proinflammatory pattern. Currently, there is great interest in the development of agents that can target senescent cells by either eliminating them or reversing the profile of their secretome (95). The relationship that the emergence of these cells has with sarcopenia and osteoporosis has yet to be determined.

## IMPACT OF MUSCLE DISEASES ON BONE

Several rare genetic diseases may present with muscle atrophy, either due to a direct muscle defect or indirect atrophy secondary to damage to the innervation system. Spinal muscular atrophy (degeneration of alpha motor neurons of the anterior horn of the spinal cord) is a group of diseases that, in their most severe forms, produce intense muscle weakness before birth and multiple contractures due to defects in joint development secondary to lack of movement, resulting in arthrogryposis multiplex congenita. Also, frequent fractures can occur both before and after birth. Duchenne muscular dystrophy, an X-linked recessive genetic disease caused by dystrophin deficiency, is also associated with bone weakness. Boys affected with this condition show progressive loss of muscle function and become unable to walk after the first decade of life. Long bone fractures are frequent and can occur even before the dystrophy is diagnosed (96). The bone condition can be worsened by glucocorticoids, which may have an important role in the emergence of vertebral fractures. Individuals with spina bifida present impaired muscle function in the lower limbs but preserved upper limb muscles (97). A previous study has found that lower limb fractures are 10 times more frequent in children who are unable to walk than in those who have this ability preserved (97).

The bone effects from muscular dystrophies that start early in life usually have a common pattern. Long bones have low bone density in the trabecular-rich regions around metaphyses and thin cortical regions in diaphyses, reflecting, respectively, trabecular catabolism and low periosteal apposition. From a clinical point of view, there is a striking difference in patterns of fractures between children who are normal versus those who have muscular dystrophy, with fractures occurring more commonly in the upper limbs in normal children and in the lower limbs in children with muscular dystrophy.

In summary, mesenchymal tissues share important structural and metabolic functions and are distinguished by broad functional plasticity. Refinements in cellular and molecular investigations have shown that these tissues play a broad role in mutual regulation. Not only their normal functions are interdependent, but diseases that primarily affect one have repercussions on the others. Muscle, adipose, and bone tissues are deeply affected by deteriorations that occur during aging and in common diseases such as DM and obesity. Although a wide window of possibilities for innovative therapies in this area has opened up, the understanding of these interactions between muscle, adipose, and bone tissues serves as a warning that potential risks of mutual effects must be carefully considered.
